# The orthotropic viscoelastic characterisation of sub-zero 3D-printed poly(vinyl alcohol) cryogel

**DOI:** 10.1557/s43580-021-00086-1

**Published:** 2021-06-22

**Authors:** J. P. Crolla, M. M. Britton, D. M. Espino, L. E. J. Thomas-Seale

**Affiliations:** 1 Dept. of Mechanical Engineering, University of Birmingham, Birmingham, B15 2TT UK; 2 School of Chemistry, University of Birmingham, Birmingham, B15 2TT UK

**Keywords:** 3D printing, Additive manufacturing, Polymer

## Abstract

**Abstract:**

Poly(vinyl alcohol) cryogel (PVA) is a versatile biomaterial used to replicate the biomechanics of tissues. Additive manufacture (AM) at sub-zero (°C) temperatures enables the manufacture of PVA with complex geometry; however, the effect of processing parameters on the mechanical properties of PVA has not been evaluated. The aim of this study is to understand the impact of print nozzle diameter and orientation on the viscoelastic mechanical properties of PVA. Samples of sub-zero AM PVA, with different filament thicknesses, were tested under tension relative to the print direction, to calculate the storage and loss moduli. As the nozzle size was decreased, AM PVA exhibited more pronounced orthotropic properties; the smallest size showed a 33% decrease in storage moduli when tested perpendicular to the print direction, as opposed to parallel. This study has demonstrated the ability of sub-zero AM to tailor the orthotropic properties of PVA.

**Graphic abstract:**

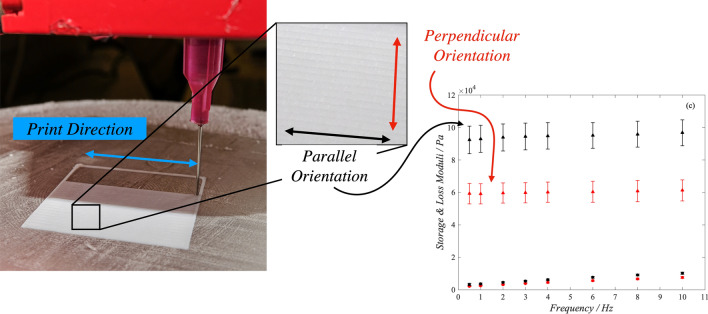

**Supplementary Information:**

The online version contains supplementary material available at 10.1557/s43580-021-00086-1.

## Introduction

Additive manufacturing (AM) is a key enabler of design for materials, particularly to replicate the complexity of bio-inspired features [1]. However, whilst AM can create enhanced geometrical complexity, there are a limited number of compatible biomaterials. The AM of connective tissue (e.g. articular cartilage and arteries) is currently constrained by a trade-off between biocompatibility and representative mechanical properties [2]. Byrne et al. (2018) propose ‘biologicalisation in manufacturing’ as a method by which bio-inspired principles can be used to develop new frontiers in digitalisation and advanced manufacturing [3]. Developing the complexity of AM platforms would allow traditional biomaterials to be manufactured additively. Drawing comparisons between developmental biology and AM, Saliba et al. (2020) propose the use of manufacturing process parameters as variables of design [4].

Polyvinyl alcohol (PVA) cryogel perpetuates research interest as a biomaterial capable of replicating the mechanical properties of tissue. Recent studies have described the development of AM, to include sub-zero (°C) temperatures or cryogenic printing platforms. Sub-zero AM has, thus, enabled the manufacture of composite PVA hydrogels through physical crosslinking upon deposition. Compressive characterisation has shown that AM of PVA composites can replicate the soft tissue mechanics of brain [5] and cartilage [6]. Furthermore, PVA itself has been shown to achieve properties akin to tendons [7]. The mechanical properties of PVA are highly dependent on freeze–thaw (FT) parameters [8]; thus, any characterisation of sub-zero 3D-printed PVA is only relevant to the reported manufacturing parameters.

PVA has long-standing applications in the mimicking of arterial tissue [9]. Yet, arteries display complex mechanical properties, including hyperelasticity, viscoelasticity, and anisotropy [10]. Ersumo et al. (2016) showed that AM changes the time-dependent mechanical properties of hydrogels [11]. Indeed, extrusion-based AM inherently creates a direction dependency relative to the toolpath. AM of PVA offers a unique opportunity to explore the direction dependency of the mechanical properties. To date, however, sub-zero AM of PVA remains mechanically uncharacterised with respect to manufacturing parameters.

Drawing on the bio-inspired concept of varying manufacturing parameters, as a method by which to achieve enhanced material complexity [4], this study aims to characterise the orthotropic viscoelastic properties of sub-zero 3D-printed PVA with varying manufacturing parameters. The dynamic viscoelastic properties of uniaxial tension characterisation shall be presented with respect to filament orientation and nozzle width.

## Materials and methods

### Sample preparation

PVA (Sigma Aldrich, St Louis, Missouri, USA), with a molecular weight of 146–186 kDa, hydrolysis of 99 + % and concentration of 10% w/w, was dissolved in deionised water by mechanically stirring at 90 °C for one hour. Stirring was continued for 1 h after removing from the hotplate, until the solution reached room temperature. After AM, all samples underwent a further three 24 h FT cycles.

### AM protocol

All samples were manufactured with dimensions of 10 × 6 mm. Sub-zero AM was conducted on an Allevi 2 Bioprinter (Philadelphia, Pennsylvania, USA). The final sample thickness was dependent on the nozzle size. Extrusion nozzles with standard needle gauges of 25G, 22G, and 18G were utilised (these will be referred to as small, medium, and large nozzles respectively), with 12 samples manufactured with each nozzle (Table 1). All samples were manufactured with a print speed of 500 mm/min. For each nozzle size, 6 samples were mechanically tested in a parallel and 6 in a perpendicular orientation (relative to the print direction). A further 6 control samples were manufactured by casting (Table 1).

**Table 1:** Manufacturing parameters and sample thicknesses for the DMA samples

	25G (small)	22G (medium)	18G (Large)	Cast
Inner nozzle diameter(mm)	0.26	0.34	0.84	N/A
Filament width (mm)	0.45	0.7	1.2	N/A
Line spacing (mm)	0.4	0.63	1.0	N/A
Pressure (kPa)	310	172	70	N/A
Sample thickness (mm)	0.5	0.75	0.9	0.75

**Fig. 1:**
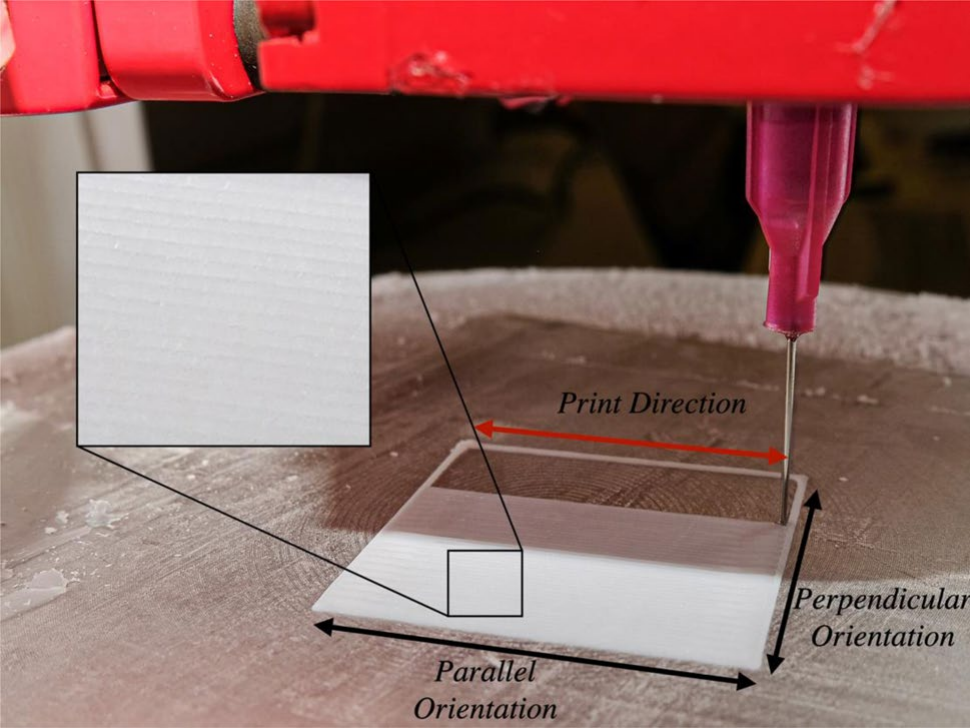
The sub-zero 3D-printing set-up for extrusion of PVA. This image includes an example of the surface morphology of the sample

Samples were extruded at a room temperature of 24 °C (± 1 °C), onto a hardened steel printing platform cooled to − 35 °C (± 1 °C) prior to the print (Fig. 1). The time-dependent temperature profile of the printing platform is presented in supplementary data. This methodology ensured the preservation of the PVA filament and overall geometry during extrusion.

For all samples, the layer height was set as equal to the inner diameter of the nozzle. In bioprinting, the pressure is not linearly proportional to the extrusion rate. The chosen printing pressure and respective filament width are shown in Table 1. The line spacing used throughout the print was set as 90% of the filament width. This line spacing ensured a balance between manufacture with no defects and no over extrusion.

To ensure that the extruded PVA was frozen immediately on contact with the printing platform, the sample thickness needed to remain less than 1 mm. Therefore, all samples printed with the small and medium syringe sizes were manufactured with two layers, and samples printed with the large nozzle size had one layer. The variability of sample thickness was taken into account in the DMA analysis (Sect. 2.3).

### Dynamic mechanical analysis

A Bose Electroforce 3200 (Bose Corporation; now TA Instruments, Delaware, USA) mechanical testing machine, running Wintest software (TA Instruments, Delaware, USA) was used to perform all DMA. This protocol has been previously used to characterise the viscoelastic properties of soft biological tissues [12–15]. Samples were tested under tension between 15 and 20% of their initial height using a frequency sweep (0.5, 1, 2, 3, 4, 6, 8, and 10 Hz). The complex $(K^{*} )$, storage $(k^{\prime})$, and loss $(k^{\prime\prime})$ stiffness were calculated by the Wintest software (Eqs. 1–3) from the fast Fourier transform of the measured sinusoidal load and displacement. Storage $(E^{\prime})$ and loss ($(E^{\prime\prime})$ moduli were then calculated relative to the shape factor ($S$) of the sample (Eqs. 4–6). Lawless et al. (2016) outline this protocol in further detail [16].

1 $$K* = ~\frac{{F*}}{{d*}}$$

2 $$k^{\prime} = k*\cos \left( \delta \right)$$

3 $$k^{\prime\prime} = k*\sin \left( \delta \right)$$

4 $$S = l~ \times w~ \times h~$$

5 $$E^{\prime} = \frac{{k^{\prime}}}{S}$$

6 $$E^{\prime\prime} = \frac{{k^{\prime\prime}}}{S}$$

### Statistical Analysis

All statistical analysis was performed using MATLAB (MathWorks, Massachusetts, USA). 95% confidence intervals were calculated. Unpaired student *t* tests were used to ascertain whether a statistically significant difference was present between the parallel and perpendicular orientations (complete datasets, including the control cast samples are shown in the supplementary information).

## Results

**Figure 2:**
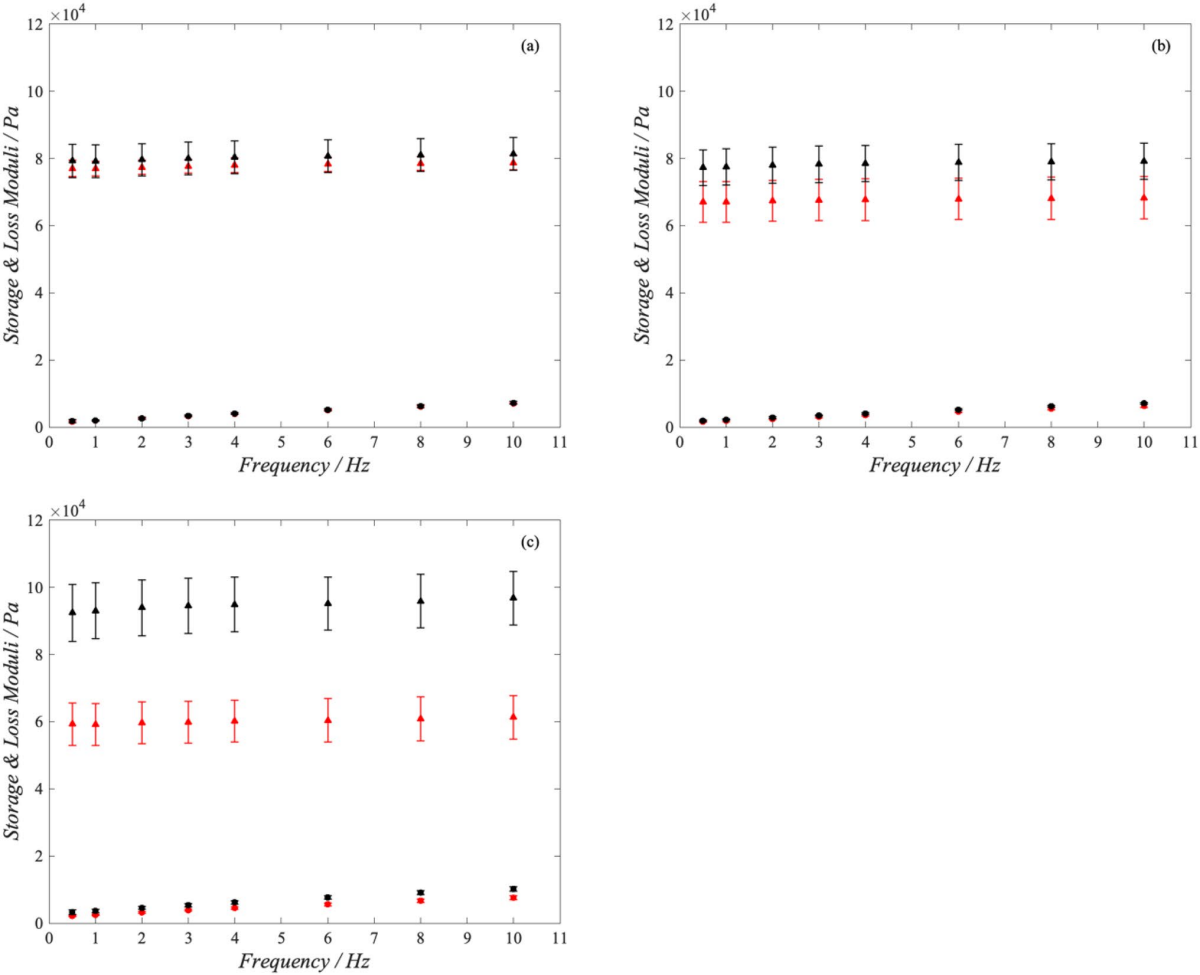
Storage (triangles) and loss (circles) moduli for AM samples using (a) 18, (b) 22G, and (c) 25G nozzle diameters. Samples tested in the parallel and perpendicular orientations are shown in red and black, respectively. Error bars show 95% confidence intervals.

Figure 1 shows an example of the surface of the samples. The toolpath-dependent meso-structure of the morphology is clearly visible. The method of manufacturing, by nature, creates mechanical properties which are dependent on direction. This is demonstrated by comparing the moduli of the parallel (red) and perpendicular (black) datasets in Fig. 2. Further to this, the orthotropic nature of additively manufactured PVA was shown to be significantly affected by the nozzle size relative to print direction (Fig. 2). Samples manufactured with the small nozzle showed an average 36.4 ± 0.26% increase in storage modulus across all frequencies when mechanically tested parallel to the print orientation (compared to perpendicular). In comparison, the medium nozzle samples demonstrated a 13.6 ± 0.18% increase in storage moduli between the parallel and perpendicular orientation. No statistically significant difference was seen for the large nozzle size.

A similar trend was seen with loss modulus (Fig. 2), with samples manufactured using the small nozzle showing the greatest increase in moduli between the parallel and perpendicular orientation. It was noted that this increase in loss moduli was also dependent on frequency, ranging from a 34.2% increase at 0.5 Hz to 25.6% increase at 10 Hz. A smaller increase was noted for samples manufactured using the medium nozzle, ranging from a 16.1% increase at 0.5 Hz to 11.0% increase at 10 Hz. No statistically significance was identified with frequency for samples manufactured with the largest nozzle.

## Discussion

This study has shown the capacity of AM to control and manufacture orthotropic mechanical properties into PVA cryogels whilst retaining material homogeneity. A change in nozzle size created a change in the viscoelastic behaviour of the cryogel, relative to the printing direction. Chen et al. (2019) demonstrated that polymer chain alignment in PVA results in an anisotropy through a process of ‘drawing’ [17]. Since PVA has previously been shown to be a shear thinning fluid [18]; the authors hypothesise that a decrease in nozzle diameter (requiring an increase in print pressure) causes further alignment of the polymer chains within the PVA solution. Whilst Millon et al. (2006 and 2007) showed that the introduction of tensile stress during the FT manufacturing process increases and allows control over the anisotropy in PVA cryogels [19, 20], this process is unlikely to retain sample geometry and is reliant on simple methods of manufacture. This study has shown another methodology to create anisotropy within PVA, which gives greater design control over the mechanical properties.

The samples which were tested in the perpendicular orientation, tended to fail along the boundaries between the filament strands. This confirms the second mechanism by which anisotropy can be introduced into the cryogel: toolpath orientation. The length of time taken for PVA for freeze has been shown to impact the number of physical crosslinks that form when undergoing FT cycles [21]. In order to retain the geometric properties of a PVA filament, there must be a short time period between deposition and freezing. However, this may also inhibit the formation of physical crosslinks between filament strands, as the PVA solution will not have as much contact time between strands of filament, before it is frozen into a cryogel. This results in weaker physical bonds between the filaments along the loading direction for the perpendicular samples. The significant decrease in storage modulus seen in the perpendicular orientation when the nozzle diameter is reduced to 0.26 mm, is due to the increased number of filament strands, and therefore, number of weak boundaries. A smaller nozzle diameter also results in extrusion of a smaller mass flow rate, thus, a reduction in thermal mass, and faster freezing time.

The results of this research have shown that AM processing parameters have a measurable impact on the mechanical properties and visible meso-scale morphology of PVA. Although the authors suggest possible mechanisms to explain the orthotropic behaviour shown, these hypotheses could be investigated further through surface imaging techniques (e.g. scanning electron microscopy) to study the morphology at a higher magnification.

This study has shown the importance of understanding all the process parameters involved in AM of PVA at sub-zero temperatures. By altering these parameters, the research has shown that it is possible to control the orthotropic behaviour, without changing the composition of PVA used, or introducing ‘post-processing’ techniques such as straining samples during the FT process.

## Conclusion

This study demonstrates that sub-zero AM can be used to control the orthotropic viscoelastic behaviour of PVA through the process parameter variation; demonstrating the future potential to design more mechanically complex connective tissue replacements.

This study has shown that for sub-zero AM of PVA:

- A decrease in nozzle size increases the direction dependence of the mechanical properties.
- Smaller nozzle sizes result in a stiffer material in the parallel orientation.
- Sub-zero 3D printing introduces ‘weak boundaries’ between extruded PVA filaments.

## Supplementary Information

Below is the link to the electronic supplementary material.

Supplementary file1 (PDF 117 kb)

## Data Availability

The datasets generated during and/or analysed during the current study are available from the corresponding author on reasonable request.
