# Supplementary material for: The orthotropic viscoelastic characterisation of sub-zero 3D-printed poly(vinyl alcohol) cryogel
Source: MRS Adv. 2021 Jun 22;6(18):467–71. doi: 10.1557/s43580-021-00086-1 (PMC8550303; doi:10.1557/s43580-021-00086-1)
Supplement: Supplementary file 1 — Supplementary file1 (PDF 117 kb) [file 43580_2021_86_MOESM1_ESM.pdf]

Supplementary Data

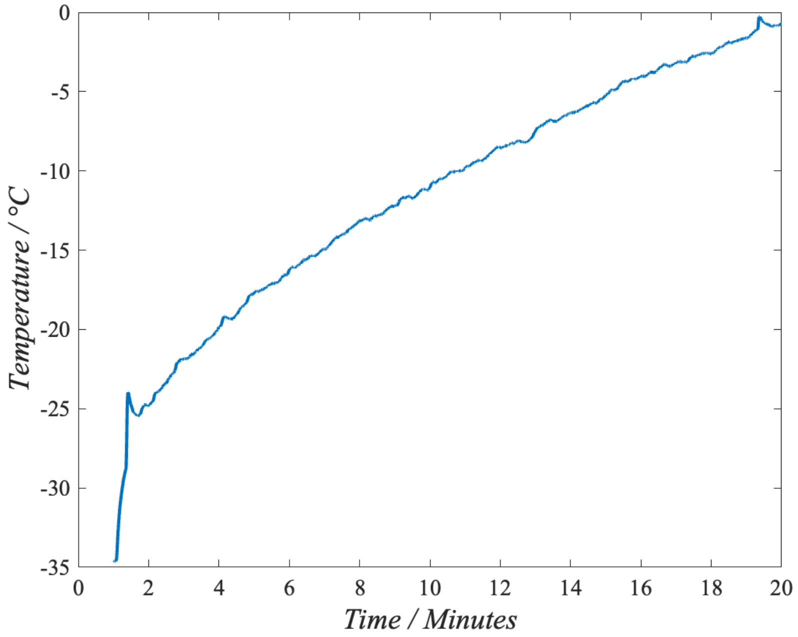

Figure 1: Temperature profile of the steel print bed during the first 20 minutes upon removal from the freezer. Samples were manufactured within the first 5 minutes of removal from the freezer.

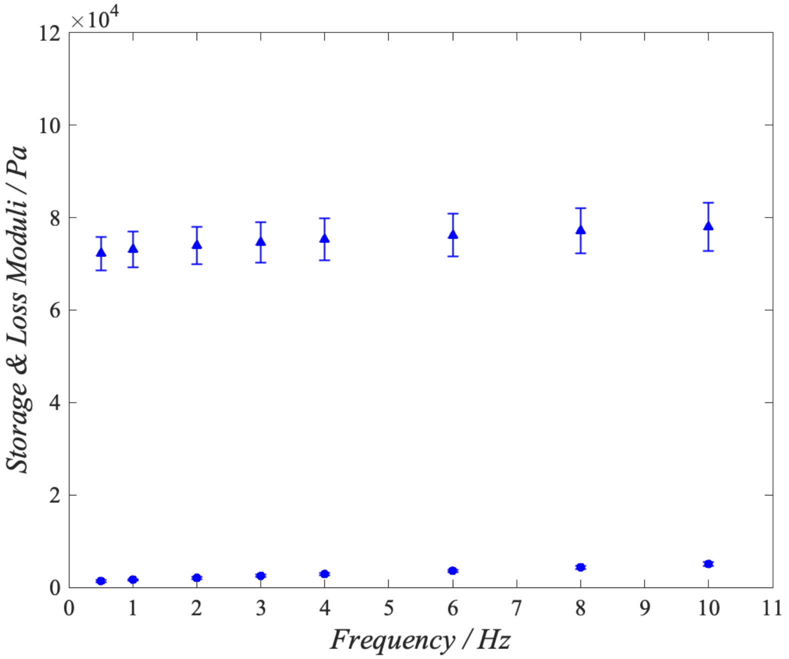

Figure 2: Storage (triangles) and loss (circles) moduli for cast control samples. Error bars show 95% confidence intervals.

	Perpendicular		Parallel	
Freq (Hz)	E' (Pa)	SD	E' (Pa)	SD
0.5	59300	5490	92400	7380
1	59200	5420	93000	7240
2	59700	5470	93900	7230
3	59900	5420	94400	7190
4	60200	5460	94900	7090
6	60400	5580	95200	6920
8	60800	5660	95900	6900
10	61300	5670	96800	6950
	E" (Pa)	SD	E" (Pa)	SD
0.5	2150	164	3260	564
1	2450	187	3650	423
2	3170	214	4540	416
3	3830	253	5350	434
4	4520	291	6220	441
6	5620	377	7650	459
8	6690	439	9080	528
10	7580	451	10200	600

Table 1: Mean Values and Standard Deviation for storage and loss moduli of additively manufactured samples of PVA using a 25G nozzle. (N = 6)

	Perpendicular		Parallel	
Freq (Hz)	E' (Pa)	SD	E' (Pa)	SD
0.5	67000	5240	77300	4640
1	67000	5240	77500	4640
2	67400	5280	78000	4620
3	67600	5340	78300	4700
4	67700	5380	78500	4680
6	68000	5380	78900	4670
8	68200	5430	79100	4700
10	68300	5490	79100	4680
	E" (Pa)	SD	E" (Pa)	SD
0.5	1570	114	1870	213
1	1850	154	2200	157
2	2430	185	2840	179
3	3020	203	3460	165
4	3550	250	4010	178
6	4620	302	5150	235
8	5480	341	6180	247
10	6310	374	7090	269

Table 2: Mean Values and Standard Deviation for storage and loss moduli of additively manufactured samples of PVA using a 22G nozzle. (N = 6)

	Perpendicular		Parallel	
Freq (Hz)	E' (Pa)	SD	E' (Pa)	SD
0.5	77100	2130	79300	4330
1	77000	1950	79200	4250
2	77400	1880	79600	4160
3	77700	1820	80000	4240
4	78000	1860	80400	4260
6	78300	1820	80700	4280
8	78500	1810	81000	4220
10	78700	1810	81300	4220
	E" (Pa)	SD	E" (Pa)	SD
0.5	1550	225	1800	420
1	1930	61.4	1960	171
2	2590	70.3	2580	295
3	3190	81.3	3340	201
4	3890	88.8	4020	248
6	5030	119	5150	286
8	6060	180	6210	368
10	7000	158	7180	375

Table 3: Mean Values and Standard Deviation for storage and loss moduli of additively manufactured samples of PVA using a 18G nozzle. (N = 6)

Freq	E' (Pa)	SD	E" (Pa)	SD
0.5	72300	2930	1360	155
1	73100	3120	1670	145
2	74000	3250	2060	179
3	74600	3500	2460	194
4	75300	3660	2890	232
6	76200	3720	3590	221
8	77100	3940	4340	288
10	78000	4160	5070	352

Table 4: Mean Values and Standard Deviation for storage and loss moduli of cast control samples of PVA. (N = 6)

	0.51 mm		0.71 mm		1.28 mm	
Freq	E'	E"	E'	E"	E'	E"
0.1	35.8	34.2	13.2	16.1	2.8	13.8
1	36.4	32.8	13.5	16.2	2.78	1.51
2	36.5	30.3	13.6	14.4	2.77	-0.538
3	36.6	28.4	13.6	12.8	2.93	4.59
4	36.6	27.3	13.8	11.4	2.98	3.23
6	36.5	26.5	13.8	10.4	3.02	2.4
8	36.6	26.3	13.8	11.3	3.1	2.42
10	36.7	25.6	13.7	11	3.21	2.57
Average	36.4	28.9	13.6	12.9	2.95	3.75
SD	0.255	2.96	0.181	2.17	0.148	4.04

Table 5: Average increase in storage and loss moduli when comparing additively manufactured samples of PVA tested in perpendicular and parallel orrientiatons for three nozzle sizes. (N = 6)

Freq (Hz)	25G (small)		22G (medium)		18G (large)	
	E' (Pa)	E" (Pa)	E' (Pa)	E" (Pa)	E' (Pa)	E" (Pa)
0.5	1.10E-05	0.0017	0.0085	0.019	0.33	0.27
1	7.90E-06	0.00018	0.0074	0.0046	0.32	0.72
2	7.30E-06	6.20E-05	0.0069	0.0053	0.3	0.92
3	6.40E-06	4.90E-05	0.0073	0.0035	0.28	0.15
4	5.80E-06	2.90E-05	0.0068	0.0076	0.28	0.3
6	5.30E-06	1.80E-05	0.0066	0.011	0.27	0.39
8	5.10E-06	1.50E-05	0.0069	0.0041	0.25	0.43
10	4.80E-06	1.50E-05	0.0072	0.0037	0.23	0.33
Average	6.70E-06	2.60E-04	7.20E-03	7.30E-03	2.80E-01	4.40E-01
SD	1.90E-06	5.20E-04	5.30E-04	4.70E-03	2.90E-02	2.30E-01

Table 6: P-values for unpaired t-tests comparing storage and loss moduli tested in perpendicular and parallel orientations for 3 nozzle sizes.
